# Co‐producing a board game to learn and engage about dementia inequalities: First impacts on knowledge in the general population

**DOI:** 10.1111/hex.13977

**Published:** 2024-01-17

**Authors:** Clarissa Giebel, Kerry Hanna, Hilary Tetlow, Mark Gabbay, Jacqui Cannon

**Affiliations:** ^1^ Department of Primary Care and Mental Health University of Liverpool Liverpool UK; ^2^ NIHR Applied Research Collaboration North West Coast Liverpool UK; ^3^ School of Health Sciences University of Liverpool Liverpool UK; ^4^ SURF Liverpool Liverpool UK; ^5^ Lewy Body Society Wigan UK

**Keywords:** dementia, diagnosis, game, inequalities, social care

## Abstract

**Background:**

Receiving and accessing care after a diagnosis of dementia, both for the person and their carer, are fraught with inequalities. The aim of this public engagement activity was to co‐produce a board game about dementia inequalities to facilitate learning, dialogue and educate about different barriers, and facilitators, to diagnosis and care and to test the game's impact on dementia knowledge with the general public.

**Methods:**

Two virtual and two face‐to‐face workshops with people with dementia, unpaid carers, health and social care professionals and Third Sector representatives were held between October 2022 and June 2023. Virtual workshops involved discussions of inequalities and how a board game may feature inequalities. The first face‐to‐face workshop was split into the same activities, aided by outcomes from workshops 1 and 2. Workshop 4 attendees tested the prototype. The impact of the game on knowledge about dementia and inequalities was tested at a game play workshop in October 2023.

**Results:**

Forty stakeholders attended four workshops. Workshops provided step‐by‐step thoughts on how the game could be designed or modified. The final game, prototype tested in workshop 4, consists of a one‐sided, two‐half board depicting the prediagnosis process (left half) and postdiagnosis process (right half). Fifty‐two members of the general public participated in the game play workshop, which led to significant improvements in knowledge about dementia (*p* < .001) and inequalities (*p* < .001).

**Discussion:**

The game can be used to improve knowledge about dementia inequalities for health and social care professionals, carers, people living with dementia, decision makers and the general public.

**Patient or Public Contribution:**

This engagement activity fully involved people with dementia, unpaid carers, health and social care professionals and Third Sector representatives throughout, with two unpaid carers as public advisers on the team.

## 1 INTRODUCTION

With an estimated 55 million people living with dementia worldwide,^1^ not everyone receives equal access to a diagnosis, or to care thereafter. The majority of people face barriers in accessing a diagnosis and are affected by further diverse inequalities in getting the care they need.^2^, ^3^

Receiving a diagnosis of dementia can be challenging, and is affected by many factors, including education, culture, stigma, general practitioner (GP) knowledge, age and dementia subtype.^4^, ^5^, ^6^ Culture and understanding of dementia can be significant barriers, especially among people from minority ethnic backgrounds in high‐income countries and people residing in low‐ and middle‐income countries (LMICs).^2^, ^7^ Dementia is often heavily stigmatised within communities, and adding to that a lack of resources for adequate healthcare, people living in LMICs face particularly serious barriers on the path to diagnosis.^8^ Regardless of where people live, being aged below 65 and/or experiencing symptoms that are part of a rarer subtype of dementia, such as behavioural‐variant frontotemporal dementia, Lewy body dementia, semantic dementia or posterior cortical atrophy, makes an individual less likely to receive a diagnosis or receive the diagnosis belatedly compared to those aged above 65 and experiencing symptoms of the most common form of dementia—Alzheimer's disease.^9^ This is linked to a lack of GP awareness of the symptoms, with GPs often failing recognising that someone in their 30s may experience symptoms of dementia.^4^

Where people do receive a diagnosis, the journey afterwards is often also characterised by various hurdles. These can include inequalities due to dementia subtype, living location, geographical differences in service provisions (‘postcode lottery’, whereby residents in adjacent postcodes or nearby streets receive different access to care due to catchment areas), availability of an unpaid carer, financial background, education, knowledge of available services and many more.^10^, ^11^, ^12^, ^13^ Accessing postdiagnostic support, such as daycare, respite care, peer support groups and paid home care, can be facilitated by financial support from local government social care services in the United Kingdom and is means tested. However, many people with dementia and their carers have to pay for care themselves or supplement their limited free care by self‐funding, as a recent 3‐UK‐nation (England, Wales, Northern Ireland) survey on the impact of the cost of living crisis on people with dementia's ability to fund social care and support services has also highlighted.^14^ While people with dementia and their carers may know they need support, many lack the knowledge of available and existing services to access within the community.^15^ Having a link person, or Dementia Care Navigator, can help facilitate improved access to care, a service that is strongly embedded within the Dutch care system for example.^10^ Having a link person that is culturally sensitive may also circumnavigate the substantial barriers that those from minority ethnic backgrounds are facing in trying to find culturally suitable services.^16^ A link person may also help those with young‐onset dementia and those with rarer subtypes of dementia, who face additional barriers to accessing care.^17^, ^18^ Thus, inequalities both for getting a diagnosis, and for living with the condition, are plentiful, yet still little addressed in practice, leading to different health and well‐being outcomes for people with dementia and their families depending on their background characteristics, as well as system‐level factors.

While standard interventions can try and address inequalities in dementia diagnosis and care as much as possible within continued social care budget restraints, a board game may raise awareness among professionals (both in training and qualified), the public, carers and people living with dementia, about these issues, generate discussion and enable sharing knowledge about barriers, facilitators and solutions. Previous board games into dementia have specifically looked at mindful storytelling^19^ and activities and storytelling related to dementia for people with dementia,^20^ while one digital game successfully raised general awareness and perception of dementia.^21^ Niedderer et al.^19^ co‐produced a board game focusing on using mindful life‐storytelling in dementia to support well‐being and quality of life. In the game, players (people with dementia) roll a dice and pick up cards with mindful questions, to reflect on different life experiences and to try and adopt a positive outlook on life. The final game was qualitatively evaluated with 50 people with dementia and 19 carers across four countries, evidencing the game's facilitation of meaningful social interaction and increased acceptance of the diagnosis. In addition, Branco et al.^20^ had co‐produced a single board game as a co‐production and participatory activity with people with dementia in two different care facilities and their families. It is unclear however what the researchers meant by ‘care facilities’. The aim of the activity was to generate activities to engage and socialise in storytelling between the family and the person with dementia, about activities taking place at the care facilities. These activities could be personalised. This game was specific to the two care institutions and the people with dementia being cared for, with the aim of replicating some of the activities engaged with at the facilities at home with the family carer. The game is based on the goose game structure, with each field representing an activity. Players need to pick up cards that provide further details on an activity of reflection. No information was provided on the number of families and people with dementia who engaged in the co‐production. Thus, while limited, very topic‐specific, and personalised board games have been co‐developed in the field of dementia, none has focused on the full trajectory of dementia (from pre‐ to postdiagnosis), associated inequalities and potential facilitators to overcoming those. A game can take the crucial step from research evidence to implementation and dissemination and is thus a very suitable format to translate knowledge in this topic area to raise awareness of dementia and associated inequalities.

The aim of this paper was to detail the methods of co‐producing a board game into dementia inequalities via public engagement and to test the impact of the game on knowledge about dementia and associated inequalities in members of the general public. This approach can be used for other research topics and evidence collections to widen impact and generate an engaging format of dissemination.

## 2 METHODS

### 2.1 Co‐production workshops and game development

**Figure 1:**
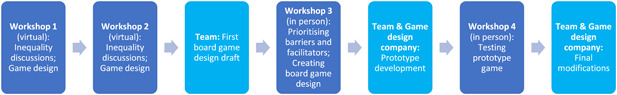
The Dementia Inequalities Game—process of game development.

A total of four co‐production public engagement workshops were held to co‐design the Dementia Inequalities Game, to translate research evidence to implementation. Figure 1 depicts the process of game development and co‐production workshops at each stage. Hosting both virtual and face‐to‐face workshops enabled wider access to participation in the co‐production and public engagement of the game development, with some unpaid carers for example having to care for their relatives at home and thus unable to attend in‐person events.

Eligible attendees included people living with dementia, current and former unpaid carers for someone with dementia, health and social care providers, Third Sector and Local Government social care representatives. Each workshop had different attendees, and co‐production and public engagement workshops were purposefully mixed to ensure a wider representation of voices and experiences. Attendees were recruited via existing networks with National Health Service, social care and Third Sector organisations, the Liverpool Dementia & Ageing Research Forum and social media. Attendees were reimbursed with a £25 shopping voucher for their time.

The first two two‐hour co‐production workshops (October 2022, January 2023) were held virtually on Zoom, involving (1) introductions of attendees and facilitators; (2) discussion of inequalities in dementia and (3) discussion of ideas for how a board game could potentially look and what it should entail. For both discussion points, a Jamboard© (digital interactive whiteboard) was used so that each attendee could either add their thoughts or the facilitator could add these during ongoing discussions. Using this approach enabled the generation of various ideas without being restricted to a limit of suggestions.

In between the virtual and face‐to‐face workshops, the team produced print‐outs of all barriers on coloured cards, distinguished by colour into pre‐ and postdiagnosis. The team also produced a basic sketch design of the board game as suggested by attendees from workshops 1 and 2 for attendees to draw on, without having had a preconceived idea of what a game should look like.

At workshop 3 (March 2023), attendees were seated at group tables, each facilitated by one or two research team members. Attendees were given two activities: (1) to prioritise different barriers for pre‐ and postdiagnosis; and (2) in the discussion, drawing onto the printed‐out board game sketches of how the basic structure of the game could be advanced. Each activity lasted approximately 50 min, with times for introductions and a refreshment break in between. All facilitators took notes of discussions at their respective group table, and summarised the discussion points at the end for the whole workshop.

After workshop 3, the research team fed the workshop discussions and suggestions from the sketch drawings onto the electronic board game design. A game developer (Focus Games) was employed to formally design the board game, and to produce four copies of a prototype.

At workshop 4 (June 2023), people living with dementia, unpaid carers, health and social care professionals, PhD students, academics and academic administrators played the prototype of the game and provided feedback. Attendees were grouped into three groups, with each consisting of between four to five players.

### 2.2 Game play workshop

In collaboration with the House of Memories, a Liverpool‐based Third Sector organisation providing training and information sessions about dementia and how to use memory objects to people with dementia, unpaid carers, and health and social care professionals, we held a 6‐h open door and prebook game play workshop at the Museum of Liverpool. 10 games were set out on tables available to play for any member of the general public aged 18+. Attendees were invited via a mailout and social media via the public Liverpool Dementia & Ageing Research Forum and the House of Memories and approached in and outside the museum with leaflets about the game play workshop. Attendees were provided with a hot drink during the workshop. If interested, attendees were provided with a study information sheet, a study consent form, and a pre‐ and postgame play dementia knowledge questionnaire. This brief two‐sided questionnaire (see Appendix 1) was co‐developed with both public advisers to have a short evaluation of the impact of game play on knowledge about dementia and associated inequalities. Pre‐ and postgame play knowledge about dementia (Question 1) and dementia inequalities (Question 2) were rated on a Likert Scale ranging from ‘1’ (poor) to ‘5’ (very good).

Attendees were able to play within their own social group, or could join other attendees. The research team was available throughout the workshop and could answer questions. However, to mimic the impact of the game play in non‐evaluation settings, the team provided no additional instructions or guidance but left attendees to read through the game instructions themselves.

We received ethical approval from the [Ref: 12878] before conducting the game play workshop.

### 2.3 Analysis of game play workshop knowledge questionnaire data

We conducted paired samples t‐tests to compare potential significant differences in pre‐ and postgame questions on dementia knowledge (Question 1) and dementia inequalities (Question 2).

### 2.4 Public involvement within the team

Two unpaid carers for former relatives with dementia (J. C., H. T.), both of whom are running their own dementia Third Sector organisation, were part of the research team. Both carers contributed to all aspects of the project, including designing and co‐facilitating the co‐production workshops, designing the board game based on workshop suggestions and feedback, as well as dissemination.

## 3 RESULTS

### 3.1 Co‐production workshops

Forty people with dementia, unpaid carers, health and social care professionals, and Third Sector representatives, as well as academics, PhD students and academic administrators (workshop 4 only) participated across the four workshops (*n*_1_ = 7; *n*_2_ = 3; *n*_3_ = 18; *n*_4_ = 12). Workshops 1 and 2 generated (1) different barriers and facilitators experienced when trying to access dementia care services; and (2) first ideas about how a board game should capture these. Across the workshops, it emerged that only focusing on postdiagnostic inequalities failed to include a key part of the dementia trajectory, and associated inequalities—namely receiving a diagnosis. Thus, a key decision emerging from the workshops was to create a double‐sided board game, with one side representing prediagnosis, and one the other side representing postdiagnosis. Table 1 lists all identified barriers pre‐ and postdiagnosis.

**Table 1:** List of identified and discussed barriers pre‐ and postdiagnosis.

Barriers prediagnosis	Barriers postdiagnosis
Unable to use digital forms of communication/support	Unsuitable support groups/activities
Sensory impairments preventing access	Care home location
Lack of funding (local area)	Unable to use digital forms of communication/support
Staff are not familiar/trained in dementia diagnosis (e.g., rare dementias)	sensory impairments preventing access
Cultural/language barrier	Lack of funding (local area)
Stigma	Staff are not equipped/trained/motivated
Postcode lottery, e.g., continuing healthcare awarded to some people in particular areas and not in others	Legalities, e.g., power of attorney
Unable to accept diagnosis	Cultural/language barrier
Mobility/transport issues	Stigma
The signs/symptoms they experience (could lead to misdiagnosis)	Postcode lottery, e.g., continuing healthcare awarded to some people in particular areas and not in others
The decision not to diagnose	Lack of information provided when diagnosed
Lack of help‐seeking behaviour (reluctance to see a professional/get a diagnosis)	Unaware that you are a ‘carer’
Rarer dementia subtype diagnosis	Anger/distressed caused by carers coming into home
Young‐onset dementia (age)	Difficulties getting care funding from the care sector and local authority)
Lack of knowledge about symptomatology	Mobility/transport issues
	Lack of support from family
	High costs associated with care
	Too much information
	Rarer dementia subtype diagnosis
	Young‐onset dementia (age)
	Lack of knowledge about symptomatology
	Not having an unpaid carer (living alone)
	Lack of link worker (Dementia Care Navigator)
	Lack of personal wealth

The fourth workshop was conducted after the prototype had been designed and focused on attendees providing feedback on the game and gaming experiences. Attendees suggested changing the dice colour to red to stand out more, adding a ‘roll‐again’ option when rolling a six on the dice, and providing further clarifications for the game instructions. Clinicians suggested removing the activities element from the cards as they were too distracting, and the activities prediagnosis might feel too similar to a cognitive assessment for people living with dementia. However, all the other players enjoyed the activities. As a result of this feedback, we have added a sentence at the bottom of each Activity card stating that if a player does not wish to engage in the activity, they can take a Question card instead.

### 3.2 The game

#### 3.2.1 Overall concept

Based on existing evidence from the research team and from further afield, and based on the two‐step co‐production process, the board game incorporates barriers and facilitators to receiving a diagnosis and postdiagnostic dementia care on a *Snake and Ladder* style format. The game comprises two halves, with one half focusing on the diagnosis process, and the other half on postdiagnostic care. On the left side of the game (diagnosis), the end point and goal of the game is to receive a diagnosis. On the right side of the game (postdiagnosis), the starting point is the diagnosis, and the endpoint depicts living well with dementia. The game is designed for 2–4 players or teams, who in turn roll a dice to move forward on the board. When a player rolls a six, they can roll the dice again.

**Figure 2:**
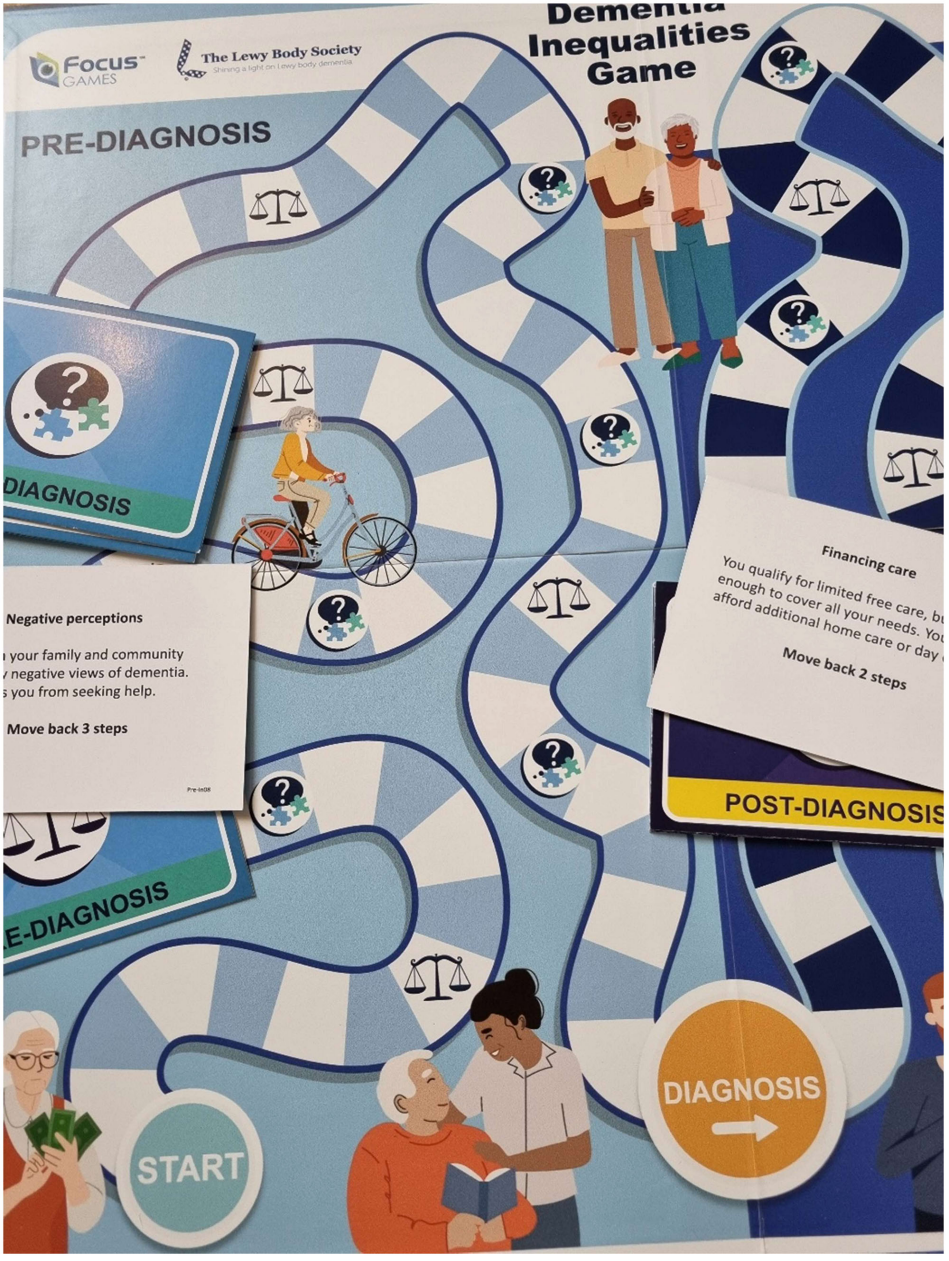
The Dementia Inequalities Game.

People depicted on the game graphics are purposefully diverse and inclusive, including gender, age and ethnic background, to address some of the underlying characteristics that can lead to inequalities in dementia. Figure 2 shows a visual of the board game and the cards.

##### Inequalities embedded within board game

Different barriers to dementia diagnosis and care were discussed and generated with workshop attendees, feeding into different inequality cards for the game. If a player moves onto a field indicated with a ‘scale’, an opposing player (or team) reads out the next card in the relevant stack. Each card includes either a barrier or facilitator to care, and asks the player to either move several steps forward (facilitator) or backwards (barrier).

##### Questions and activities

Players can move onto fields where they are asked to pick up a ‘Question or Activity’ card. When a player chooses the correct answer from the three options offered, or undertakes the activity, they move forward one step. If they answer the question incorrectly, they stay on their current game spot.

For the prediagnosis side of the game, activities were derived from memory assessment tools used in the diagnosis process of dementia, such as the Mini‐Mental State Examination,^22^ the Montreal Cognitive Assessment,^23^ and the Addenbrooke's Cognitive Examination III.^24^ For example, players might be asked to remember 5 digits. On the postdiagnosis board game side, activities focused on engaging in activities that are shown to improve well‐being in dementia and linked to delayed symptom deterioration. For example, players might be asked to do a wall‐sit, thus referring to physical fitness and activity in general.

### 3.3 Impact of the game on knowledge about dementia and inequalities

Fifty‐seven members of the general public participated in the game play workshop, with five attendees not completing the postgame questionnaire or handing in their questionnaire. Thus, 52 members of the general public aged 18+ participated and completed the questionnaire. Participants were on average 46 years old (±18) (range 19–83), and were predominantly female (*n* = 30; 57.7%).

Paired samples *t* test showed a significant difference in pre‐ and postgame play knowledge about dementia [*t*(51) = −3.470, *p* < .001], with a pregame mean score of 3.04 (±1.15) and postgame of 3.5 (±0.9). Paired samples *t* test also showed a significant difference in pre‐ and postgame knowledge about inequalities in dementia [*t*(50) = −6.268, *p* < .001], with a pregame mean score of 2.39 (±1.08) and postgame of 3.35 (±1.07). Thus, playing the game improved both knowledge of dementia, and dementia inequalities.

## 4 DISCUSSION

This is the first game to focus on inequalities in dementia diagnosis and care, which has been co‐produced at all stages of its development. The final game version includes content demonstrating various inequalities both in the prediagnosis and postdiagnosis stages, as well as additional questions to raise general awareness about dementia. The game can also be seen as a boundary object, prompting discussions about health inequalities, associated stigma, barriers and solutions to overcome them along the dementia journey. Playing the game can successfully improve knowledge about dementia and associated inequalities.

Without any previous co‐produced game on inequalities in dementia, this public engagement activity and quantitative evaluation of the Dementia Inequalities Game has shown that the game fills a necessary and innovative gap to educate about the topic and reach nonacademic stakeholders (including health and social care professionals, students, people affected by dementia) and the general public. This game has translated a substantial evidence base on dementia inequalities, via in‐depth co‐production, and this paper provides a framework for translating evidence in other research areas into a game. Previous games focused on mindful storytelling^19^ and activities and storytelling related to dementia for people with dementia,^20^ and our game provides an informative and engaging approach to real‐life barriers to diagnosis and care different to the existing developed games. Future work needs to focus on co‐producing a theory of change, to explore how engaging with the Dementia Inequalities Game can impact on knowledge and potential care practices in health and social care professionals. This can be guided by delivering game play workshops to professionals with key principles identified as part of dementia education programmes by Phillipson et al.^25^ These include using simple and clear messages and outcomes for the game and providing incentives to play the game. These have been identified for healthcare professionals, based on using Knowledge Translation as a conceptual framework for general learning. However, these principles could also be applied to social care professionals (including those working in home care, daycare, respite care, and residential care). Given the severe restraints on many health and social care professionals' time and high job demands, conducting a 1‐h educational, and social, game play workshop, can provide an important opportunity to learn and socialise with peers in the workplace environment, without the need to travel to take individual training courses.

## 5 CONCLUSION

This is the first fully co‐produced game on dementia inequalities, with early evidence from the general public indicating its successful impact on improving knowledge about dementia and associated inequalities. The next step will be to run a full evaluation of the impact of the game play on knowledge about dementia and associated inequalities with health and social care professionals and students, possibly with more in‐depth dementia knowledge questionnaires such as the Dementia Knowledge Assessment Scale,^26^ which was considered too lengthy for this pilot general public evaluation of a drop‐in game play workshop. It is also important to assess whether engaging in the game may change access to dementia diagnosis and care in the long term for people living with dementia and their carers and can lead to changes to care delivery in the health and social care workforce. In the future, the Dementia Inequalities Game has the potential to be modified for different countries or regions across the world, to be used as an awareness‐raising and education tool to overcome stigma and improve knowledge.

## AUTHOR CONTRIBUTIONS

**Clarissa Giebel**: Conceptualisation; investigation; funding acquisition; writing—original draft; methodology; writing—review and editing; formal analysis; project administration; data curation. **Kerry Hanna**: Conceptualisation; investigation; methodology; data curation; writing—review and editing. **Hilary Tetlow**: Conceptualisation; investigation; data curation; writing—review and editing. **Mark Gabbay**: Conceptualisation; investigation; funding acquisition; writing—review and editing; data curation. **Jacqui Cannon**: Writing—review and editing; conceptualisation; investigation; data curation.

## CONFLICT OF INTEREST STATEMENT

Hilary Tetlow is the organiser of SURF Liverpool, which funded the fourth workshop. The remaining authors declare no conflict of interest.

## ETHICS STATEMENT

The authors received University of Liverpool ethics approval [Ref: 12878] before the game play workshop.

## Data Availability

The data that support the findings of this study are available from the corresponding author upon reasonable request.

## APPENDIX I DEMENTIA KNOWLEDGE QUESTIONNAIRE

**Dementia Inequalities Game Evaluation**

**Pregame Questionnaire**

(please circle your responses and write in the provided text boxes)

1. How would you rate your knowledge about dementia? (1—poor → 5—very good)
  **Table:**
  1	2	3	4	5
2. How would you rate your knowledge about inequalities in dementia?
  **Table:**
  1	2	3	4	5
3. Do you know how many people live with dementia in the UK?
4. Do you know the most common type of dementia?

**Postgame Questionnaire**

(please circle your responses and write in the provided text boxes)

1. How would you rate your knowledge about dementia?
  **Table:**
  1	2	3	4	5
2. How would you rate your knowledge about inequalities in dementia?
  **Table:**
  1	2	3	4	5
3. Do you know how many people live with dementia in the UK?
4. Do you know the most common type of dementia?
5. What have you learned from playing this game?
6. Will you make any changes as a result of your learning from this game? This could be in your clinical practice or daily life.
